# The Rwandan Healthcare System: Can a Shifting Burden of Disease Threaten a Post-war Success Story?

**DOI:** 10.7759/cureus.53957

**Published:** 2024-02-10

**Authors:** Iyesatta M Emeli

**Affiliations:** 1 Emergency Department, Emory University, Atlanta, USA

**Keywords:** healthcare financing, post-war, social determinants of health, sub-saharan africa, universal health care, low middle income countries, post-conflict, rwanda

## Abstract

Rwanda is located in Central Africa, bordered by the Democratic Republic of Congo (DRC), Burundi, Tanzania, and Uganda. In 1994, Rwanda was immersed in a brutal war and genocide. Rwanda’s subsequent remarkable post-war recovery has been well documented. What this paper aims to do is to explore Rwanda’s successes and the vulnerability it faces with the shifting burdens of diseases. This paper seeks to contribute to the global discourse on effective healthcare models in resource-limited, post-conflict settings, even as such countries achieve improved socio-economic conditions and experience associated changes in population disease patterns.

## Editorial

Introduction

Rwanda is located in Central Africa, bordered by the Democratic Republic of Congo (DRC), Burundi, Tanzania, and Uganda. It is small and densely populated (26,331 sq. km - slightly smaller than Maryland; population size 12,461,00 (2019 estimate) [1], over 400 people/sq. km [2] and it is the fifth most densely populated country in the world [3]). The 1994 Rwandan genocide left 800,000 people dead in 100 days and many displaced and wounded [4].

Rwanda’s subsequent remarkable post-war recovery in the domain of healthcare as measured by healthcare outcomes such as child mortality, life expectancy, and achievement of the Millennium Development Goals for health has been well described [5,6].

This paper seeks to explore the achievements this country has made with its universal health care model in the aftermath of such great tragedy and loss and to highlight how the country’s successes are not accidental but born out of deliberate multi-level policies to address not only health care access but also social determinants of health and the forces and systems that shape the wellbeing of a population. Included is an overview of Rwanda’s health finance model. Importantly, success can bring new sets of challenges; with economic development there is an associated shift in burdens of disease, a change termed the epidemiologic transition [7] and this paper will explore this potential vulnerability that any post-conflict resource-poor country can expect to face if and when they are able to achieve economic success and improved healthcare outcomes.

The 1994 Rwandan genocide

The 1994 Rwandan genocide left 800,000 people dead and, in 1994, to be a Rwandan was to have a life expectancy of just 29 [8,9]. The profound social injustice in Rwanda was evident in poor healthcare outcomes, early death, and low life expectancy of the population. Neighboring Uganda and DRC fared better at the time (life expectancy of 44 and 49 respectively) [9], though obviously still much worse than Western and North American countries’ life expectancies.

By 2016, however, Rwanda had a greater life expectancy than its neighbors (67 [8] compared to Uganda’s and DRC’s life expectancy of 60) [9]. This was the result of deliberate interventions that improved not only healthcare access and infrastructure but also sought to improve social determinants of health. The circumstance and lived experience of the average Rwandan have changed markedly since 1994 and Rwanda continues to enjoy political stability and economic growth. With an emphasis on homegrown policies and solutions, Rwanda has prioritized access to services and education and improved living conditions for its citizens [10].

Social determinants of health

War and armed conflict come with death, maiming, gender-based violence, displacement, catastrophic effects on mental and physical health, depletion of social resources, destruction of infrastructure, and debilitating loss of human capital. This leads to immediate health disparities with long-term consequences. This was the scenario Rwanda faced and it reflected poor health outcomes. The advent of political stability and socio-economic growth has led to a marked improvement in the same outcomes.

Life expectancy doubled in the 20 years post the 1994 genocide as a result of policies to increase the number of health facilities, improve health coverage, and ensure access to safe drinking water and good housing [10,11]. These policies also saw Rwanda become one of only two sub-Saharan African countries to meet all the health Millennium Development Goals (MDG) with a marked decline in under-five mortality rates and improvement in maternal mortality [10].

Shifting the burden of disease

The burden of disease is a measure of mortality and morbidity; of what kills and what ails a given population. With economic development, there is a change in the character and pattern of disease; a shift from communicable, maternal, neonatal, and nutritional (CMNNDs) to non-communicable diseases (NCDs) and lifestyle-related chronic illness; this change is called the epidemiologic transition [7].

**Figure 1:**
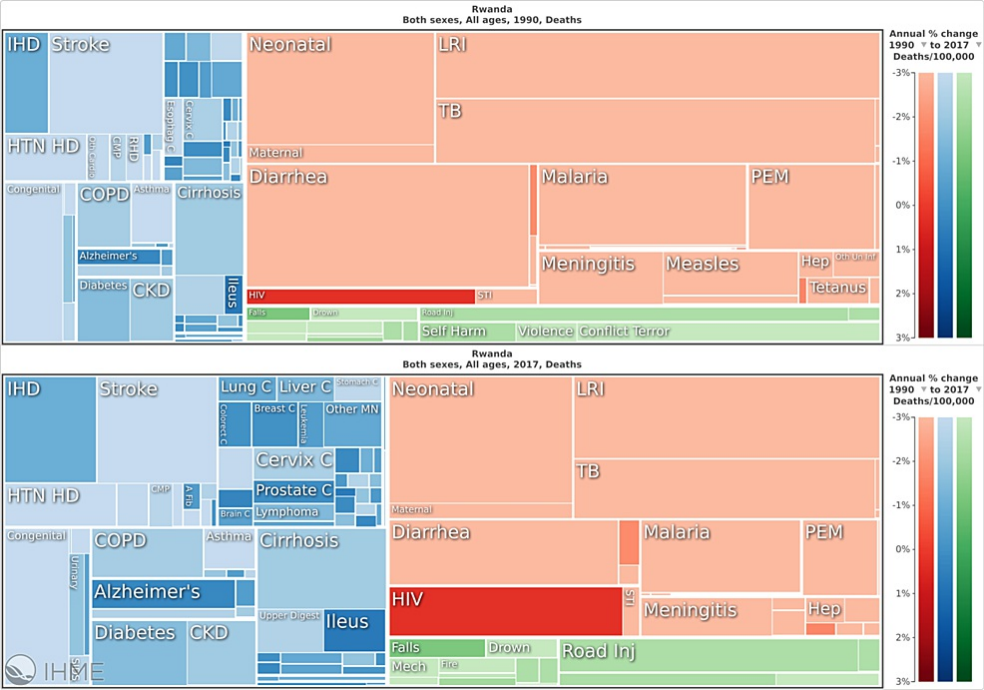
Shifting burden of disease in Rwanda, 1990 vs 2017. Blue and green represent deaths due to non-communicable diseases (NCDs) and Injuries, respectively. Red represents deaths due to communicable, maternal, neonatal and nutritional (CMNNDs). Source: Institute for Health Metrics and Evaluation. Used with permission. All rights reserved [12].

**Figure 2:**
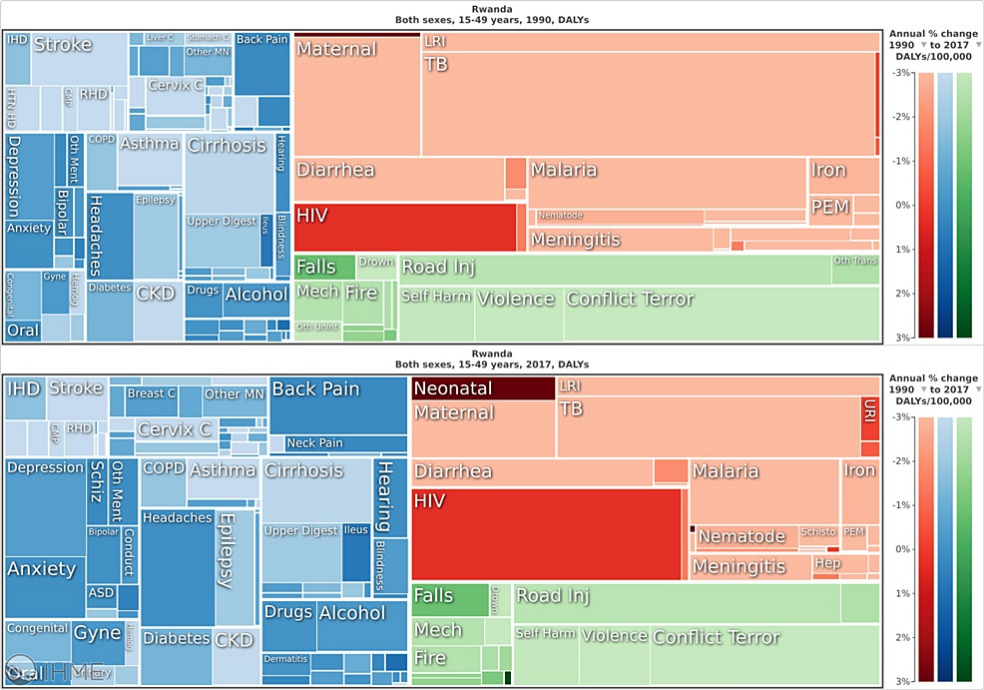
Shifting burden of disease in Rwanda, 1990 vs 2017. Blue and green represent disability adjusted life years (DALYs) due to non-communicable diseases (NCDs) and Injuries respectively. Red represents DALYs due to communicable, maternal, neonatal and nutritional (CMNNDs). Source: Institute for Health Metrics and Evaluation. Used with permission. All rights reserved [12].

Figures 1, 2 provide a visual of the transition showing an increasing dominance of blue and green (NCDs and Injuries) as compared to red (CMNNDs) from 1990 to 2017 with respect to death and DALYs (disability adjusted life year- measure of years lost to both death and disability) in Rwanda [12]. With increasing socio-economic status and the government’s continued investments in public and preventative health, the shift will continue moving Rwanda from a period where CMNNDs dominate, to a period of double burden of both, to then an NCD-dominant picture.

This shift in burden of disease is highly relevant to Rwanda’s health financing model and can present an area of vulnerability.

Health financing in Rwanda

Rwanda has a universal healthcare model, and 90% of the population is enrolled in a community-based health insurance scheme (with sliding scale premiums adjusted for income) called Mutuelles de Santé [13].

Any healthcare plan and expenditure should aim at broad and affordable coverage and minimize out-of-pocket expenditure of the citizens for preventive and basic healthcare. By eliminating such financial barriers to care, people are incentivized to seek continued preventive care and early care in the setting of acute illness thereby reducing costs to the system while also improving health outcomes. Rwanda’s Mutelles de Santes is achieving this. Drawbacks cited about the system are the limitations in the depth of coverage [14] and affordability for the poorest citizens. The premiums, which were initially US$ 2 per family member per annum but now vary on a sliding scale according to family income/wealth, can still be a significant financial barrier to the most vulnerable citizens [15].

The government, the citizens, Muteulle’s premiums, out-of-pocket expenses, developmental assistance, charity organizations, NGOs, and development partners pay for Mutuelles funding with approximately 50% of Mutuelles’ funding coming from annual member premiums [13]. This model, however, still involves a dependence on development assistance for health. 2016 health financing data from the Institute for Health Metrics and Evaluation (IHME) indicated that $19 out of $44 per person health care expenditure came from development assistance [12]. This dependence is an area of vulnerability. In particular, dependence on donors who prioritize CMNND investments creates a situation wherein NCDs are neglected. As such, citizens spend a disproportionate amount of out-of-pocket expenses on NCDs. It has been found that over half of spending on cardiovascular disease in low-income countries has been out-of-pocket for patients [16].

Conclusion

Rwanda continues to enjoy strong economic growth and adherence to strategic policies that increase access to healthcare, improve living standards, and address social determinants of health. The country now aims to achieve middle-income country status by 2035 and high-income country status by 2050 [10]. Even after the global economic strain of the COVID-19 pandemic, Rwanda’s economy showed resilience registering real GDP growth of 8.2% in 2022 [10]. With this continued improvement in socioeconomic status, there will also continue to be shifts in burdens of disease from CMNNDs to NCDs. Rwanda will need to ensure it has a healthcare plan and healthcare financing model to address such shifts. Irrespective of these challenges and potential vulnerabilities, Rwanda stands as a model of what can be achieved even in the aftermath of brutal conflict and extreme tragedy.
